# Validation of myocarditis diagnoses in the Swedish patient register for analyses of potential adverse reactions to COVID-19 vaccines

**DOI:** 10.48101/ujms.v128.9290

**Published:** 2023-05-11

**Authors:** Rolf Gedeborg, Lennart Holm, Nils Feltelius, Anders Sundström, Kai M Eggers, Marja-Leena Nurminen, Maria Grünewald, Nicklas Pihlström, Björn Zethelius, Rickard Ljung

**Affiliations:** a Department of Efficacy and Safety 1, Division of Licensing, Medical Products Agency, Uppsala, Sweden; b Department of Surgical Sciences, Uppsala University, Sweden; c Office of Use and Information, Division of Use and Information, Medical Products Agency, Uppsala, Sweden; d Department of Drug Safety, Division of Use and Information, Medical Products Agency, Uppsala, Sweden; e Department of Medical Sciences, Uppsala University, Uppsala, Sweden; f Statistics group, Department of Efficacy and Safety 2, Division of Licensing, Medical Products Agency, Uppsala, Sweden; g Institute of Environmental Medicine, Karolinska Institutet, Stockholm, Sweden

**Keywords:** COVID-19 vaccines, myocarditis, diagnosis, validation study

## Abstract

**Background:**

Coronavirus disease 2019 (COVID-19) mRNA vaccines are associated with an increased risk of myocarditis using hospital discharge diagnoses as an outcome. The validity of these register-based diagnoses is uncertain.

**Methods:**

Patient records for subjects < 40 years of age and a diagnosis of myocarditis in the Swedish National Patient Register were manually reviewed. Brighton Collaboration diagnosis criteria for myocarditis were applied based on patient history, clinical examination, laboratory data, electrocardiograms, echocardiography, magnetic resonance imaging and myocardial biopsy. Poisson regression was used to estimate incidence rate ratios, comparing the register-based outcome variable to validated outcomes. Interrater reliability was assessed by a blinded re-evaluation.

**Results:**

Overall, 95.6% (327/342) of cases registered as myocarditis were confirmed (definite, probable or possible myocarditis according to Brighton Collaboration diagnosis criteria, positive predictive value 0.96 [95% CI 0.93–0.98]). Of the 4.4% (15/342) cases reclassified as no myocarditis or as insufficient information, two cases had been exposed to the COVID-19 vaccine no more than 28 days before the myocarditis diagnosis, two cases were exposed >28 days before admission and 11 cases were unexposed to the vaccine. The reclassification had only minor impact on incidence rate ratios for myocarditis following COVID-19 vaccination. In total, 51 cases were sampled for a blinded re-evaluation. Of the 30 randomly sampled cases initially classified as either definite or probably myocarditis, none were re-classified after re-evaluation. Of the in all 15 cases initially classified as no myocarditis or insufficient information, 7 were after re-evaluation re-classified as probable or possible myocarditis. This re-classification was mostly due to substantial variability in electrocardiogram interpretation.

**Conclusion:**

This validation of register-based diagnoses of myocarditis by manual patient record review confirmed the register diagnosis in 96% of cases and had high interrater reliability. Reclassification had only a minor impact on the incidence rate ratios for myocarditis following COVID-19 vaccination.

## Introduction

A large study based on nationwide health registers in Denmark, Finland, Norway and Sweden has demonstrated that both first and second doses of mRNA Coronavirus disease 2019 (COVID-19) vaccines are associated with an increased risk of myocarditis and pericarditis (1). The primary outcome was a hospital discharge diagnosis indicating myocarditis in the respective country’s Patient Register. It is important to determine the accuracy of these diagnoses to support the validity of the estimated associations between mRNA COVID-19 vaccine exposure and myocarditis.

Clinically, the diagnosis of acute myocarditis is based on symptoms (mainly acute chest pain), electrocardiogram (ECG), echocardiography, serum biomarkers for myocardial injury, magnetic resonance imaging (MRI) and/or endomyocardial biopsy (2). There are no previous international consensus case definitions for myocarditis as adverse events following immunisation. Such criteria have, however, recently been proposed by the Brighton Collaboration (3).

The aim of this study was to validate the accuracy of a myocarditis diagnosis when based on ICD-10 hospital discharge diagnoses in the Swedish National Patient Register, applying the proposed Brighton Collaboration criteria in a structured manual review of patient records.

## Methods

### Study population

To enable pharmacoepidemiological studies of the COVID-19 vaccines, the Swedish Medical Products Agency has set up a regularly updated dynamic nationwide register-based study cohort (CoVacSafe-SE). This research database has been generated from individual-level linkage of COVID-19 vaccination exposure data to other national health data registers (4). It primarily included all individuals permanently residing in Sweden on 31 December 2020 and has been continuously updated with, e.g. information on exposure to COVID-19 vaccines and diagnoses from the Swedish National Patient Register.

For validation of ICD-10 diagnoses of myocarditis, registered in the Swedish National Patient Register and linked to CoVacSafe-SE, we selected individuals 12–39 years old having an incident hospital discharge diagnosis of myocarditis identified during the study period from 27 December 2020 to 13 November 2021. These selection criteria are identical to those in the Nordic study on SARS-CoV-2 vaccination and myocarditis (1) but cover a longer time period. All cases of myocarditis with a prior exposure to the COVID-19 vaccine and a random selection of cases unexposed to the COVID-19 vaccine were eligible for the validation procedure.

Our primary validation was restricted to individuals 12–39 years old because young men and adolescent boys appeared to be at the highest risk in the Nordic study (1). Individuals with any record of myocarditis from inpatient or specialised outpatient hospital care from 1 January 2017 to 26 December 2020 were considered as prevalent cases and therefore excluded from the study population.

The study was approved by the Swedish Ethical Review Authority.

### Register-based diagnosis of myocarditis

From CoVacSafe-SE cases of myocarditis were identified from primary or secondary hospital discharge codes (ICD-10-SE) I400, I401, I408, I409, I411, I418 or I514 after an in-hospital stay. Diagnoses in CoVacSafe-SE originate from exact person-based linkage to the Swedish National Patient register using the unique personal identification number (5). This register is maintained by the National Board of Health and Welfare and reporting to the register of all in-hospital care and out-patient specialist care is mandated by law. During the study period, discharge diagnoses were coded according to the Swedish clinical modification of the 10th revision of the International Statistical Classification of Diseases and Related Health Problems (ICD-10-SE) (6).

### Patient record review

Patient records covering the hospital separation where a myocarditis diagnosis occurred first were requested from that specific hospital department. Brighton Collaboration criteria for the level of evidence were applied based on patient history, clinical examination, laboratory data, electrocardiograms, echocardiography, magnetic resonance imaging and myocardial biopsy to verify the diagnosis of myocarditis. A structured electronic questionnaire was developed to capture the basic information required for the application of the Brighton Collaboration criteria for the level of evidence (see variable list in Table S1) (3). According to these criteria, each case reviewed was classified as (1) ‘Definitive case’, (2) ‘Probable case’, (3) ‘Possible case’, (4) having ‘Insufficient information’ or (5) ‘Not a case’. The Brighton Collaboration levels of evidence is an ordinal scale, and the consequences of reclassifications are different depending on where on the scale the reclassification occurs. A pragmatic case definition based on the Brighton Collaboration levels would be either level 1–2 (definite or probable) or level 1–3 (definite, probable, or possible). Each patient record was reviewed by one of four raters. All four had several years of experience from working with pharmacovigilance at the Swedish Medical Products Agency. Three were medical doctors specialised in rheumatology/internal medicine, anaesthesiology/critical care and pharmacology, respectively. One had a clinical background as anaesthesia nurse and research nurse. Effective blinding to vaccination status was deemed unfeasible. If the rater was uncertain how to interpret clinical information or classify a case, a contracted senior cardiologist at the Uppsala University Hospital was consulted. If there remained uncertainty, the case was discussed in the group of raters to reach consensus.

### Interrater reliability

To evaluate interrater reliability, we randomly selected 15 records (or all if fewer were available) from each Brighton Collaboration level for re-evaluation in a new round of patient record review. The same group of raters was used, but each rater could only reassess patient records reviewed by another rater in the first round of manual record review. Also in this second round of review, the contracted senior cardiologist could be consulted at the discretion of the individual rater.

### Statistics

Based on the results of the patient record review we described the extent and pattern of reclassification and calculated the positive predictive value of a register-based diagnosis with 95% confidence interval (CI).

To determine the sensitivity of risk estimates in the previously published Nordic myocarditis study (1) to the reclassification of the outcome, we compared the results of using the register-based outcome definition to an outcome definition requiring Brighton Collaboration level of evidence of at least *possible* (level 1–3) or *probable* (level 1–2). As only a random selection of myocarditis cases unexposed to the COVID-19 vaccine was validated, a random selection of unexposed myocarditis cases not validated was reclassified proportionate to the reclassification in the validated subset. Starting follow-up on 27 December 2020, we used Poisson regression to estimate incidence rate ratios (IRRs) with 95% CIs, comparing rates of myocarditis in 28-day risk periods after the administration date of the first and second dose of a COVID-19 vaccine to rates in unvaccinated periods, using time-varying exposures. Identical modelling of covariates as in the Nordic study was used (1).

## Results

Out of 404 cases of myocarditis 12–39 years old identified during the study period, patient records were requested for all 157 exposed cases and for a random sample of 191, out of the 247 cases unexposed to the COVID-19 vaccine. Patient records could be retrieved for 154 (98.1%) of the exposed cases and 188 (98.4%) of the randomly selected cases unexposed to the COVID-19 vaccine (Figure S1). In this population, 273 were males and 69 were females. Overall, unexposed cases tended to be more non-specific with a more complex clinical presentation. A lower proportion had elevated markers for myocardial damage and in more than half of these cases, no results from echocardiography were available (Table 1). The characteristics of the randomly selected unexposed cases were comparable to the source population of unexposed cases (Table S2).

**Table 1:** Case severity by exposure status.

Clinical severity measures	Exposed to COVID-19 vaccine ≤ 28 days before admission for myocarditis *N* = 106		Exposed to COVID-19 vaccine > 28 days before admission for myocarditis *N* = 48		Not exposed to COVID-19 vaccine before admission for myocarditis *N* = 188	
**Deaths**	0		0		0	
**Length of hospital stay** (days), median (IQR)	3	(2–4)	2	(1–4)	3	(1.75–4)
**Troponin I/T**, *n* (%)						
Elevated^a^	100	(94.3)	45	(93.8)	166	(88.3)
Missing	0		0		6	(3.2)
**Ejection fraction**, *n* (%)						
< 40	0		3	(6.3)	7	(3.7)
40–54	18	(17.0)	6	(12.5)	19	(10.1)
≥ 55	46	(43.4)	20	(41.7)	61	(32.4)
Missing	42	(39.6)	19	(39.6)	101	(53.7)

a The cut-off for elevated Troponin was analysis- and hospital-specific.

### Reclassification after patient record review

After the patient record review, 95.6% (327/342) of the cases registered as myocarditis in the Swedish National Patient Register were confirmed as having myocarditis (definite, probable or possible), while 4.4% (15/342) were reclassified as not having myocarditis (Table 2). Of these, two cases had been exposed to the COVID-19 vaccine no more than 28 days before the myocarditis diagnosis, two cases were exposed >28 days before admission and 11 cases were unexposed to vaccine. The overall positive predictive value was 0.96 (95% CI 0.93–0.98), identical when restricted to males and 0.94 (95% CI 0.86–0.98) when restricted to females. An even stricter case definition of myocarditis (definite or probable) yielded a positive predictive value of 0.94.

**Table 2:** Classification in Brighton Collaboration levels of diagnostic certainty for myocarditis from the primary patient record review, by exposure status.

Brighton Collaboration diagnostic certainty level	Exposed to COVID-19 vaccine ≤ 28 days before admission for myocarditis (*N* = 106)		Exposed to COVID-19 vaccine > 28 days before admission for myocarditis (*N* = 48)		Not exposed to COVID-19 vaccine before admission for myocarditis (*N* = 188)	
Definitive case, *n* (%)	44	(41.5)	20	(41.7)	74	(39.4)
Probable case, *n* (%)	57	(53.8)	26	(54.2)	100	(53.2)
Possible case, *n* (%)	3	(2.8)	0	(0)	3	(1.6)
Insufficient information, *n* (%)	0	(0)	1	(2.1)	3	(1.6)
Not a case, *n* (%)	2	(1.9)	1	(2.1)	8	(4.3)

### Impact of reclassification on risk estimates

**Figure. 1:**
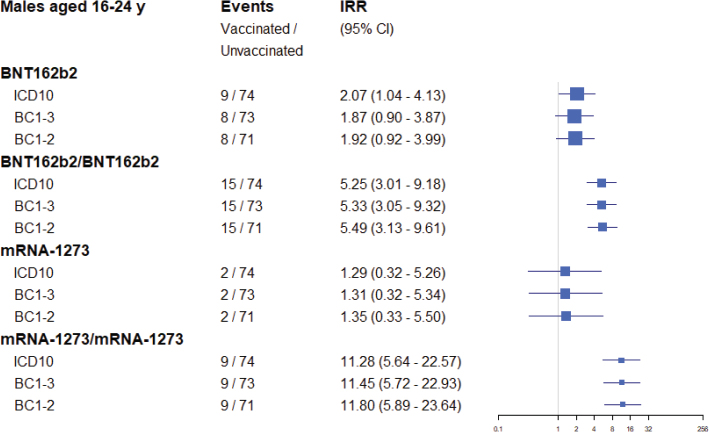
Estimated association between COVID-19 vaccines and myocarditis events within 28 days of exposure in the age group 16–24 years, comparing different definitions of the outcome variable. Squares represent incidence rate ratios with lines representing 95% confidence intervals, and arrows truncation of these intervals. A single vaccine name indicates first dose of that vaccine (eg, BNT162b2) and the risk of the outcome after the first dose. Vaccine names in combination indicate a vaccine schedule of first dose of the first vaccine and a second dose of the second vaccine (eg, BNT162b2, BNT162b2) and the risk of the outcome after the second dose. The Poisson regression model adjusted for age group and sex, previous SARS-CoV-2 infection, health care worker status, nursing home resident, and comorbidity variables.

**Figure. 2:**
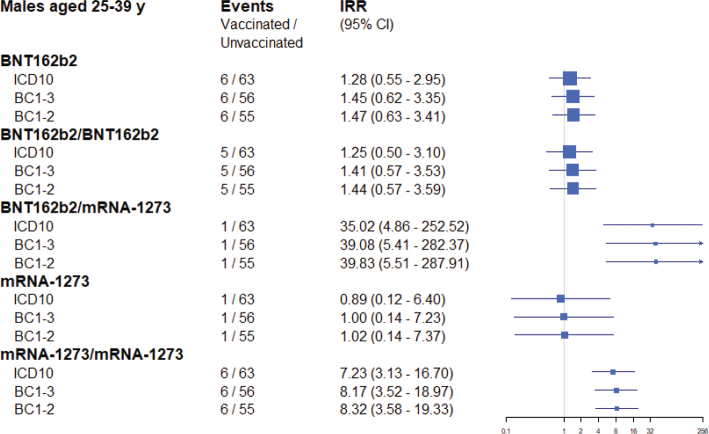
Estimated association between COVID-19 vaccines and myocarditis within 28 days of exposure in the age group 25–39 years, comparing different definitions of the outcome variable. Squares represent incidence rate ratios with 95% CIs. A single vaccine name indicates first dose of that vaccine (eg, BNT162b2) and the risk of the outcome after the first dose. Vaccine names in combination indicate a vaccine schedule of first dose of the first vaccine and a second dose of the second vaccine (eg, BNT162b2, BNT162b2) and the risk of the outcome after the second dose. The Poisson regression model adjusted for age group and sex, previous SARS-CoV-2 infection, health care worker status, nursing home resident, and comorbidity variables.

When the regression analysis to estimate the risk for myocarditis associated with COVID-19 was rerun in the age group 16–24 years old, with a case definition requiring a Brighton Collaboration level of evidence of at least *possible*, the results were only marginally different compared to using the original register diagnosis (Figure 1). An even stricter case definition, requiring a Brighton Collaboration level of evidence of at least *probable*, produced essentially identical results. A similar pattern was seen in the analysis of the age group 25–39 years old (Figure 2).

### Interrater reliability

In total, 51 cases were sampled for a blinded re-evaluation (Table 3). Of the 30 randomly sampled cases initially classified as either definite or probable myocarditis none were re-classified outside the Brighton Collaboration levels of evidence of at least *probable* (level 1–2). In all, 21 cases were initially classified as Brighton Collaboration level 3–5, of which all were sampled for re-evaluation. Among these 21 cases, eight out of the 12 reclassifications by a new rater had a direct impact on the binary outcome variable. After re-evaluation 12 of these 21 cases were classified within the Brighton Collaboration levels of evidence of at least *possible* (level 1–3).

**Table 3:** Interrater reliability analysis of myocarditis diagnosis when classified according to Brighton Collaboration level. Reclassification table for 51 patient records randomly selected (15 for each Brighton Collaboration level, or all available if fewer) from the primary validation. Re-evaluation of Brighton Collaboration level was done in a second round of patient record review by another rater blinded to the first assessment.

Brighton collaboration level from FIRST round of patient record review	Brighton collaboration level from SECOND round of patient record review				
	1. Definitive case	2. Probable case	3. Possible case	4. Insufficient information	5. Not a case
1. Definitive case	14	1	0	0	0
2. Probable case	1	14	0	0	0
3. Possible case	0	3	2	0	1
4. Insufficient information	0	1	1	1	1
5. Not a case	0	3	2	0	6

## Discussion

Approximately 4% of the cases registered as myocarditis in the Swedish National Patient Register during the study period were reclassified as not having myocarditis. The proportion reclassified was slightly higher in the group unexposed to the COVID-19 vaccine compared to those exposed to the vaccine. When the risk for myocarditis related to COVID-19 vaccines was estimated, differences in the strength of association were minor when using an outcome measure based on the patient record review, compared to using the original register diagnosis.

Before the COVID-19 pandemic, Swedish national register data have indicated a slightly increasing trend in the background incidence of myocarditis in subjects aged ≥16 years during the period 2000–2014 (7). During a 1-year follow-up, 6.4% were newly diagnosed with either heart failure or dilated cardiomyopathy. The frequency of severe outcomes was higher in older patients and occurred in the immediate post-discharge period. During the pandemic, myocarditis has been described as a rare cardiovascular complication to both the COVID-19 infection and COVID-19 vaccines (1, 8, 9). Using a register-based diagnosis of myocarditis may, however, raise concerns regarding the validity of the diagnosis. Validation is therefore important to support interpretation of findings in register-based studies.

There are currently no internationally accepted consensus criteria for myocarditis. We used the recently proposed Brighton collaboration criteria as the basis for the manual patient record review (3). Other criteria used, such as those issued by the US Centers for Disease Control and Prevention (CDC), are similar but not identical (10).

It may be difficult to apply myocarditis criteria to information extracted from patient records. Endomyocardial biopsy results are considered as a key component but are rarely justified in cases of uncomplicated myocarditis and has limited sensitivity (11). In our validation, only few patients were subjected to biopsy. This diagnostic criterion is therefore in reality of low value for studies using data from routine care. The reporting in routine patient records of results from cardiac magnetic resonance imaging (CMR) is not always easily matched to published CMR criteria for myocarditis (12). Echocardiographic results may be borderline and subjective. Findings on the ECG may be unspecific and difficult to interpret. Clinical symptoms are not systematically reported.

It is therefore important to look for interrater variability in a validation based on patient record review such as in the present study. The patient record review was not straightforward. Interrater reliability was very high in the Brighton Collaboration level 1 and level 2. However, interrater reliability was much lower in the 21 cases initially classified as level 3 to level 5, most likely related to the evaluation of electrocardiograms. This is not unexpected as substantial variability in ECG interpretation has been observed for physicians at all training levels, even after educational interventions (13–15). Several ratings changed after re-evaluation by a new rater, which in a proportion of cases also changed the value of the binary myocarditis variable. In general, the re-evaluation mostly changed the value from a non-case to a more definite case (definite, probable or possible case). The application of the Brighton Collaboration level of evidence criteria for myocarditis should be done after careful training of raters and with good support during the review process, but it may still be expected to generate some interrater variability. The assessment of patient cases with limited or borderline support for the myocarditis diagnosis is a challenge both in the clinical context and in a patient record review such as in our study. Importantly, our study did not identify any major concern with using the myocarditis diagnosis registered in the Swedish National Patient Register as outcome variable in epidemiological studies. If interrater variability has affected the estimated positive predictive value, it is likely to have resulted in an under-estimation of the quality of the registrer diagnosis.

In a previous single-centre validation 507 electronic case records with a discharge diagnosis of myocarditis were systematically reviewed, and 421 (83.0%) could be verified as acute myocarditis (7). The evaluation also of false negatives requires wider sampling criteria. In a US validation of CDC’s Vaccine Safety Datalink case criteria, they were found suboptimal by not including the ICD-10 diagnosis I51.4 (myocarditis unspecified) (16). The reduced sensitivity noted with the 15-day risk period in the CDC criteria compared to a 30-day risk period is, however, no concern for our study, since we applied a 28-day risk window.

Our study has some notable limitations. The patient record review was not blinded to vaccine exposure status. It was considered unfeasible to reliably blind the reviewers. Redaction of the extracts from patient records would signal vaccination status or if too extensive threaten the overall clinical assessment of the case. Furthermore, our study design does not allow evaluation of potentially false negative cases as this would require a large sample of health-care contacts with plausible symptoms but without a diagnosis of myocarditis in the Patient Register. This limits the ability to generate estimates of sensitivity and specificity, which would have been helpful for quantitative bias analysis. Some caution must also be exerted before generalisation to other countries since coding practices for hospital discharge diagnoses may differ, and to age groups not represented in our study population.

## Conclusion

This validation of register-based diagnoses of myocarditis by manual patient record review confirmed the register diagnosis in 96% of cases and had high interrater reliability. Among the few cases not classified as definite or probable interrater reliability was much lower, mostly due to substantial variability in electrocardiogram interpretation. Reclassification had only minor impact on the incidence rate ratios for myocarditis following COVID-19 vaccination.

## Disclosure statement

Dr Sundström reported participating in research funded by governmental agencies, universities, Astellas Pharma, Janssen Biotech, AstraZeneca, Pfizer, Roche, (then) Abbott Laboratories, (then) Schering-Plough, UCB Nordic and Sobi, with all funds paid to Karolinska Institutet, outside the submitted work.

Dr Grünewald reported being involved in the European Medicines Agency regulatory assessment of Comirnaty; being previously employed at a drug development consultancy firm with cross-product responsibilities and being involved on a project for pertussis vaccines funded by Sanofi Pasteur, Merck Sharp & Dohme Corp, and GlaxoSmithKline at the Swedish Agency of Infectious Disease Control.

Dr Ljung reported receiving grants from Sanofi Aventis paid to his institution outside the submitted work and receiving personal fees from Pfizer outside the submitted work.

## Funding

This research was conducted as a pharmacovigilance activity by the Medical Products Agency, which is a Swedish Government agency. The study did not receive any external funding.

## ORCID

Rolf Gedeborg https://orcid.org/0000-0002-8850-7863

Rickard Ljung https://orcid.org/0000-0002-0654-4530

Björn Zethelius https://orcid.org/0000-0002-1738-0834

Anders Sundström https://orcid.org/0000-0003-2337-3371

Kai Eggers https://orcid.org/0000-0002-8806-5778

Nils Feltelius https://orcid.org/0000-0003-1460-4078

Maria Grünewald https://orcid.org/0000-0002-9280-8140
