# Supplementary material for: Validation of myocarditis diagnoses in the Swedish patient register for analyses of potential adverse reactions to COVID-19 vaccines
Source: Ups J Med Sci. 2023 May 11;128:10.48101/ujms.v128.9290. doi: 10.48101/ujms.v128.9290 (PMC10202079; doi:10.48101/ujms.v128.9290)
Supplement: Supplementary file 1 [file UJMS-128-9290-s001.pdf]

Supplementary material

Figure S1. Flow chart describing the extraction of patient records for manual review from the entire source population in CoVacSafe-SE[1] for the period 27 Dec 2020 to 13 Nov 2021.

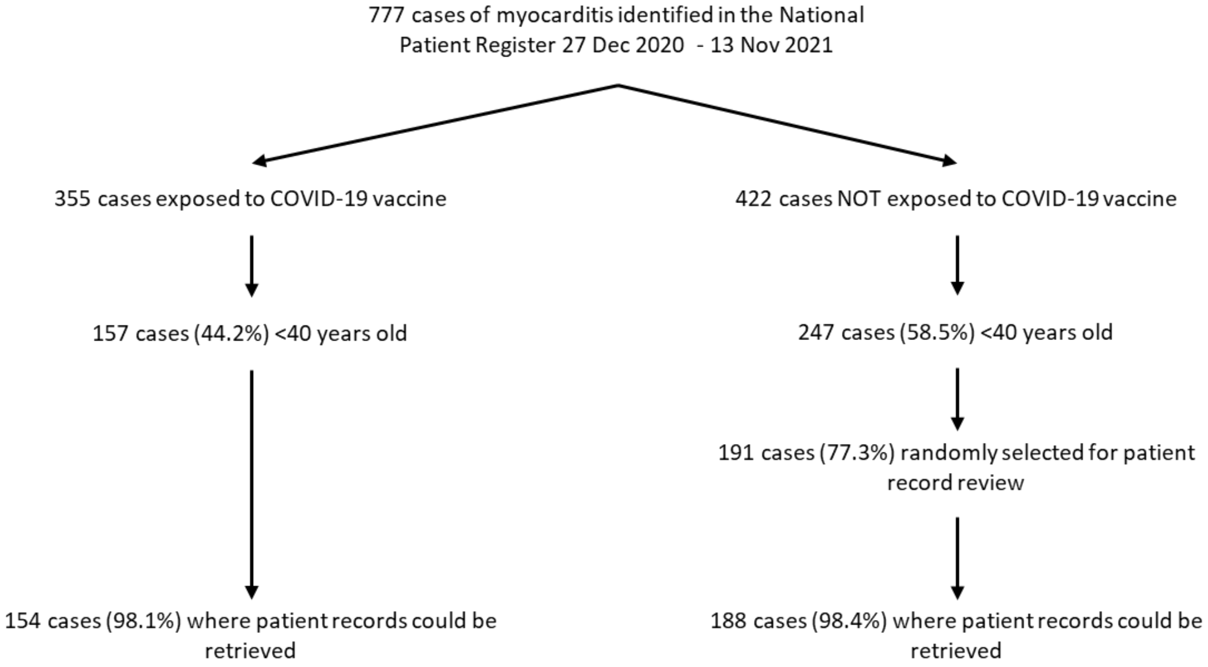

Table S1. Variables recorded in the questionnaire during manual patient chart review. The recorded symptoms, signs, findings are related both to myocarditis and pericarditis.

Sex
Age at admission
Vaccine dose 1-3
Date for start of symptoms
Admission date
Discharge date
Discharge diagnosis
Triggering event
Acute chest pain or pressure
Palpitations
Dyspnea after exercise, at rest, or lying down
Diaphoresis
Sudden death
New onset cardiac chest pain or pressure
Palpitations
Dyspnea after exercise, at rest, or lying down
Abdominal pain
Dizziness/syncope
Cough
Oedema
Fatigue
Weakness
Gastrointestinal - nausea, vomiting diarrhoea
Shoulder/upper back pain
Cyanosis
Low grade intermittent fever
Altered Mental Status
Troponin T, max value
Date Max Troponin-T
Elevated Troponin-T (Y/N)
Troponin I, max value
Date Max Troponin-I
Elevated Troponin I (Y/N)
NT-proBNP, max value
Elevated NT-proBNP (Y/N)
CRP Max
Elevated CRP (Y/N)
ESR Max
Elevated ESR (Y/N)
D-Dimer Max

Elevated D-Dimer (Y/N)
LPK Max
TPK Max
Anti-SARS-CoV-2 IgG-N (Y/N)
Anti-SARS-CoV-2 IgG-S (Y/N)
SARS-CoV-2 (Y/N)
SARS-CoV-2 test date
Any **alternative diagnosis** for symptoms
If Yes, which diagnosis?
ECG, date
Paroxysmal or sustained atrial or ventricular arrhythmias (premature atrial or ventricular beats, and/or supraventricular or ventricular tachycardia, interventricular conduction delay, abnormal Q waves, low voltages)
AV nodal conduction delays or intraventricular conduction defects (atrioventricular block (grade I-III), new bundle branch block)
Continuous ambulatory electrocardiographic monitoring that detects frequent atrial or ventricular ectopy
ST-segment or T-wave abnormalities (elevation or inversion)
PACs and PVCs
Diffuse concave-upward ST-segment elevation
ST-segment depression in aVR
PR-depression throughout the leads without reciprocal ST-segment changes
Non-specific changes that are new and/or normalize in recovery
Echocardiography, date
New focal or diffuse left or right ventricular function abnormalities (eg. decreased ejection fraction)
LV Ejection fraction (vid diagnostic Echo)
Segmental wall motion abnormalities
Global systolic or diastolic function depression/abnormality
Ventricular dilation
Wall thickness change
Intracavitary thrombi
MR, date
Oedema on T2 weighted study, typically patchy in nature
Late gadolinium enhancement on T1 weighted study with an increased enhancement ratio between myocardial and skeletal muscle typically involving at least one non-ischemic regional distribution with recovery (myocyte injury).
**Pericardial exudation (PEX)**<br>Evidence of abnormal fluid collection or pericardial inflammation by imaging (Echo, MR, cMR, CT)
**Chest radiography**<br>Chest radiography showing enlarged heart
Pericardial friction rub
Pulsus paradoxus
Distant heart sounds (infant/children)

Histopathologic examination of myocardial tissue?

Table S2. Characteristics of myocarditis patients unexposed to COVID-19 vaccine, comparing those selected for validation to the entire source population of unexposed cases with myocarditis or pericarditis in CoVacSafe-SE[1] for the period 27 Dec 2020 to 13 Nov 2021.

	Myocarditis cases selected for validation		Source population of unexposed myocarditis cases	
	**(n=191)**		**(n=247)**	
**Age**, n (%)				
12-15 years	17	(8.9)	21	(8.5)
16-24 years	84	(44)	112	(45.3)
25-39 years	90	(47.1)	114	(46.2)
**Sex**				
Males, n (%)	145	(75.9)	200	(81)
**Admission date for myocarditis**, n (%)				
dec 2020-jan 2021	21	(11)	28	(11.3)
February	19	(9.9)	28	(11.3)
March	27	(14.1)	30	(12.1)
April	14	(7.3)	19	(7.7)
May	20	(10.5)	26	(10.5)
June	21	(11)	26	(10.5)
July	21	(11)	33	(13.4)
August	13	(6.8)	16	(6.5)
September	16	(8.4)	19	(7.7)
October	16	(8.4)	17	(6.9)
November	3	(1.6)	5	(2.0)

References

1. Ljung R, Sundström A, Grünewald M, et al. The profile of the COvid-19 VACcination register SAFEty study in Sweden (CoVacSafe-SE). Upsala journal of medical sciences 2021;126.
